# Power for tests of interaction: effect of raising the Type I error rate

**DOI:** 10.1186/1742-5573-4-4

**Published:** 2007-06-19

**Authors:** Stephen W Marshall

**Affiliations:** 1 Department of Epidemiology, School of Public Health, University of North Carolina at Chapel Hill, Chapel Hill, North Carolina, USA

## Abstract

**Background:**

Power for assessing interactions during data analysis is often poor in epidemiologic studies. This is because epidemiologic studies are frequently powered primarily to assess main effects only. In light of this, some investigators raise the Type I error rate, thereby increasing power, when testing interactions. However, this is a poor analysis strategy if the study is chronically under-powered (e.g. in a small study) or already adequately powered (e.g. in a very large study). To demonstrate this point, this study quantified the gain in power for testing interactions when the Type I error rate is raised, for a variety of study sizes and types of interaction.

**Methods:**

Power was computed for the Wald test for interaction, the likelihood ratio test for interaction, and the Breslow-Day test for heterogeneity of the odds ratio. Ten types of interaction, ranging from sub-additive through to super-multiplicative, were investigated in the simple scenario of two binary risk factors. Case-control studies of various sizes were investigated (75 cases & 150 controls, 300 cases & 600 controls, and 1200 cases & 2400 controls).

**Results:**

The strategy of raising the Type I error rate from 5% to 20% resulted in a useful power gain (a gain of at least 10%, resulting in power of at least 70%) in only 7 of the 27 interaction type/study size scenarios studied (26%). In the other 20 scenarios, power was either already adequate (n = 8; 30%), or else so low that it was still weak (below 70%) even after raising the Type I error rate to 20% (n = 12; 44%).

**Conclusion:**

Relaxing the Type I error rate did not usefully improve the power for tests of interaction in many of the scenarios studied. In many studies, the small power gains obtained by raising the Type I error will be more than offset by the disadvantage of increased "false positives". I recommend investigators should not routinely raise the Type I error rate when assessing tests of interaction.

## Background

Quantification of effect-measure modification (hereafter called "modification") is an important aspect of epidemiologic research [1]. During data analysis, assessment of modification often involves testing the statistical significance of one or more interactions terms in a regression model, or using a test such as the Breslow-Day test for heterogeneity of the odds ratio [1-4].

However, power for assessing interaction during data analysis is often poor in epidemiologic studies, which are frequently designed primarily for the assessment of main effects only (the term "main effect" refers to any variable not involved in an interaction). Researchers who are reluctant to "miss" an important interaction due to low power can elect to use a higher Type I error rate when assessing interactions. A error rate of 20%, rather than the traditional 5%, has been suggested [4]. A higher Type I error rate boosts the statistical power, however, the gain in power comes at the cost of more Type I errors (spurious false positive tests for interaction). Proponents of raising the Type I error rate argue that it is preferable to include additional spurious interactions in the analysis, rather than mistakenly overlook a "true" interaction [4].

There are two main drawbacks to the strategy of increasing the power of the interaction test by raising the Type I error rate that are explored in this paper. The first problem occurs if power is at an extremely low level when the Type I error rate is 5% (e.g. if the study was very small), the power gain obtained from increasing the Type I error rate may not be large enough to boost power to an acceptable level. In this situation, the power might be slightly improved, but will still be very low, at the higher Type I error rate. In this paper, I refer to this chronically under-powered situation as the "low ground" scenario.

On the other hand, the second problem can occur if the study power is already high enough to detect an interaction of substantive importance. In this situation, there is no real need to boost power, and the effect of raising the Type I error rate is merely to dilute the pool of identified interactions by including a higher proportion of interactions that are of little substantive interest. I refer to this already-adequately-powered situation as the "high ground" scenario.

There is "middle ground" between the "low ground" and "high ground" scenarios. In the "middle ground" scenario, it makes sense to raise the Type I error rate when assessing interactions, because this will usefully boost power from a sub-standard level to a useful level.

The purpose of this study is to quantify the size of the "middle ground". In other words, how often does raising the Type I error rate for interaction tests result in a useful gain in power? If epidemiologic studies are frequently in the "middle ground", there may be a case for universally recommending that the Type I error rate routinely be raised when assessing interactions. On the other hand, if few epidemiologic studies fall into the "middle ground", then recommendations suggesting that Type I error rate be raised [4] are ill-advised and should be discontinued.

### Practical illustration of raising the Type I error rate

Assume an epidemiologist has conducted a study that assessed multiple exposures and is analyzing the data using a series of logistic regression models, some of which contained interactions. S/he is reviewing computer output that reports measures of effect (such as odds ratios) along with their confidence intervals and p-values. Under a "test-based paradigm", s/he will identify a main effect with a p-value above 5% as less predictive of the outcome than a main effect with a p-value below 5%. However, some epidemiologists, attuned to the fact that power for interactions is typically much lower than power for main effects, might elect to raise the Type I error rate to 20% when assessing interactions [4]. They would identify an interaction term with a p-value above 20% (not 5%) as a potential modifier of effect.

Of course, an extensive literature advises epidemiologists to consider measures of effect, confidence intervals, stratum-specific measures and *apriori *biological knowledge, in addition to considering p-values, when determining strength of association or assessing modification [1,5-8]. Furthermore, multiplicative models (such as logistic regression) make it difficult to assess interactions on the basis of departure from additivity of effects [1,9,10]. However, despite their limitations, p-values and multiplicative models remain a staple of modern epidemiology.

Epidemiologists frequently report p-values from tests of interaction, however, my anecdotal impression is that most epidemiologists do not raise the Type I error rate in the manner described above when testing for interactions. To quantify the frequency of the practice of using tests of interaction with a relaxed Type I error rate, I reviewed of all papers published between November 2004 and October 2005 in the American Journal of Epidemiology that included the word "interaction" in the title, abstract, or text of the paper. A total of 94 substantive papers were identified that presented some form of quantitative assessment of effect-measure modification. Of these, six papers used tests of interaction with a raised Type I error rate [11-16]. Three papers used an error rate of 10% [11-13] and three papers used an error rate of 20% [14-16]. The remaining 88 used either the standard 5% error rate or else did not report any interaction p-values.

## Methods

### Overview

The outcome of interest in this study was the gain in power obtained by raising the Type I error rate (see Appendix for definition of Type I and Type II error). Power was quantified at four Type I error rates: 5%, 10%, 15%, and 20%. The gain in power due to raising the Type I error rate was studied for ten different hypothetical types of interaction, ranging from sub-additive through to super-multiplicative, across three study sizes. Three commonly used tests of interaction were examined: the Wald test, the likelihood ratio test, and the Breslow-Day test. These tests are described in detail in the Appendix.

In the interests of simplicity, the study was focused on case-control studies of two binary exposures. The two binary exposures are referred to as exposure A and exposure B. The standard regression analysis for this data involves fitting a logistic model:

logit(D = 1) = *β*_0 _+ *β*_1_*A *+ *β*_2_*B *+ *β*_3_*AB *(model 1)

where D is coded to 0 for controls and 1 for cases, A and B are binary variables with the non-exposed coded to 0 and the exposed coded to 1, and AB is the product-term interaction obtained by multiplying A by B.

Power for tests of interactions in case-control studies has previously been examined in a study that focused on comparing additive and multiplicative models [3]. However, the previous study did not address the question of how much power is gained when the Type I error rate is raised (i.e. it presented results at the 5% level only). In order to preserve comparability with the previous work [3], I elected to make many of the parameters examined in this study (the interaction scenarios, the study sizes, the exposure prevalences, and the case:control ratio) the same (or similar) to those used by the previous author [3].

### Types of interaction

**Figure 1:**
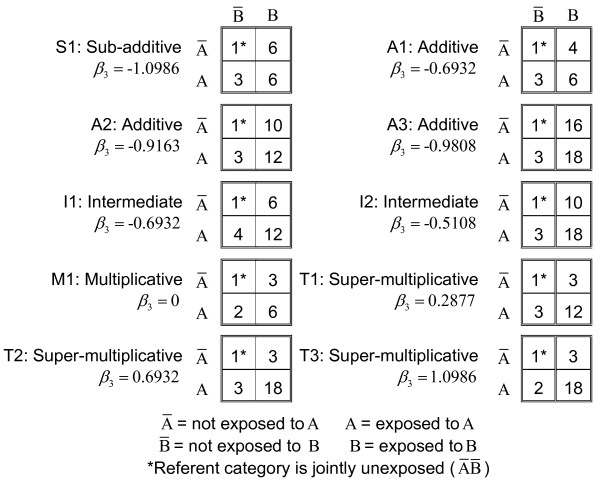
Population Odds Ratios for Ten Hypothetical Interaction Scenarios (based on Greenland, 1983).

This study examined ten different types of interaction, ranging from joint effects that were less than additive through to greater than multiplicative. These ten scenarios, described in Figure 1, cover the gamut of interactions typically encountered in epidemiology. Figure 1 shows the 2 × 2 interaction tables for the ten hypothetical source populations (not studies) [3].

Each interaction is characterized by the expected joint effect under assumptions of perfect additivity, or multiplicativity, of effects. For example, for the sub-additive interaction (S1), the joint effect of double exposure has an odds ratio of 6, which is less than the joint effect expected under perfect additivity (3 + 6 - 1 = 8). For the first super-multiplicative interaction (T1), the joint effect of double exposure is 12, which is greater than expected under perfect multiplicativity (3 × 3 = 9). The exposures in the M1 interaction are perfectly multiplicative (3 × 2 = 6) and perfectly additive in the A1, A2, and A3 interactions. Intermediates I1 and I2 are greater than additive but less than multiplicative.

### Study size and exposure prevalence

I examined studies of three different sizes: 75 cases and 150 controls (small), 300 cases and 600 controls (large), and 1200 cases and 2400 controls (very large). The case:control ratio was fixed at 1:2.

The exposure prevalences examined was also fixed. The exposure prevalence in the non-cases was set at 40% for A and 40% for B, with 40% of the non-cases being exposed to both A and B (doubly exposed), 20% being exposed to A but not to B, 20% being exposed to B but not to A, and 20% unexposed to both A and B (doubly unexposed). The doubly unexposed was the reference category in all analyses. These exposure prevalences ensured adequate numbers of cases and controls in all strata [3].

### Methods for studying power

This study used two methods for studying power: the asymptotic power function and simulations. The asymptotic power function was used to examine the power of the Wald test for the interaction in the logistic model (model 1). The term "asymptotic power" refers to the power results obtained from a formula derived under the assumption the study size is large (see Appendix).

In addition, simulations were used to confirm the asymptotic results, investigate the coverage of the 95% confidence interval, and extend the results to the likelihood ratio test for interaction and the Breslow-Day test for heterogeneity of the odds ratio. Simulation is a computer-intensive method for empirically studying the properties of a statistic by mimicking the process of conducting a large number of studies. Ten hypothetical populations were created – one for each of the ten types of interaction studied (Figure 1). One thousand with-replacement samples were drawn from each population and the three tests of interaction were computed for each sample. Each sample is, in essence, a simulated epidemiologic study. For each of the ten types of interaction, I tabulated the proportion of the 1,000 simulated samples where the p-value for interaction was significant at the 5%, 10%, 15%, and 20% level.

### Definition of a "useful gain in power"

For the purposes of this study, a useful gain in power was deemed to have occurred if: 1) power increased by at least 10% when the Type I error rate was raised from 5% to 20%, and, 2) power reached at least 70% or higher at a Type I error rate was raised of 20%. For the power gain to be less than 10%, power must be high (above 85%) at the 5% Type I error rate. Obviously this criteria is somewhat subjective and is intended as a guide to the interpretation of the results. It should not be seen as a definitive statement about the utility of raising the Type I error rate in a particular study.

The perfectly multiplicative (M1) scenario was excluded from consideration when classifying the power gains as useful or not useful. In scenario M1, the value of *β*_3 _in the underlying population is zero. Because of sampling variability, however, some M1 samples will return a positive test for a non-zero *β*_3_. These are false positive tests for interaction. The power function collapses to the Type I error rate (the false positive error rate of 5%, 10%, 15%, or 20%) when data from scenario M1 is analyzed using model 1.

## Results

### When the Type 1 error rate is 5%

Asymptotic power results for the Wald test of interaction are shown in Table 1. As expected, power was highest for those types of interaction that departed most strongly from perfect multiplicativity of effects. If the standard Type I error rate of 5% was used, power for almost all types of interaction was extremely low when the study size was 75 cases and 150 controls. For studies with 300 cases and 600 controls, the power was higher but was still typically below the range (typically 80% or better) that would be acceptable if one was designing a study to assess interaction. With 1200 cases and 2400 controls, power was at design levels for almost all types of interaction.

**Table 1:** Effect of Raising the Type I Error Rate on the Statistical Power for the Wald^1 ^Test of Interaction for Three Study Sizes in a Case-Control Study of Two Binary Exposures^2^

		Small Study Size (75 Cases & 150 Controls)				Large Study Size (300 cases & 600 controls)				Very Large Study Size (1200 Cases & 2400 Controls)			
		
Type of Interaction		Type I Error Rate				Type I Error Rate				Type I Error Rate			
		
		5%	10%	15%	20%	5%	10%	15%	20%	5%	10%	15%	20%
S1	Sub-additive	43%	55%	63%	69%	*95%*	*97%*	*98%*	*99%*	*>99%*	*>99%*	*>99%*	*>99%*
A1	Additive	21%	31%	39%	45%	**63%**	**74%**	**80%**	**84%**	*>99%*	*>99%*	*>99%*	*>99%*
A2	Additive	26%	37%	45%	52%	**75%**	**84%**	**88%**	**91%**	*>99%*	*>99%*	*>99%*	*>99%*
A3	Additive	24%	35%	43%	49%	**71%**	**81%**	**86%**	**89%**	*>99%*	*>99%*	*>99%*	*>99%*
I1	Intermediate	18%	28%	35%	42%	**55%**	**67%**	**75%**	**79%**	*99%*	*99%*	*>99%*	*>99%*
I2	Intermediate	11%	18%	24%	30%	28%	40%	48%	54%	**79%**	**87%**	**91%**	**93%**
M1	Multiplicative	5%	10%	15%	20%	5%	10%	15%	20%	5%	10%	15%	20%
T1	Super-multiplicative	7%	13%	19%	24%	14%	22%	29%	35%	40%	53%	61%	67%
T2	Super-multiplicative	16%	25%	32%	38%	**47%**	**60%**	**67%**	**73%**	*96%*	*98%*	*99%*	*99%*
T3	Super-multiplicative	30%	42%	50%	57%	**82%**	**89%**	**93%**	**95%**	*>99%*	*>99%*	*>99%*	*>99%*

^1^In simulations, almost identical results were obtained for the Likelihood Ratio test and Breslow-Day Test

^2^**Bolded **table cells indicate "middle-ground" scenarios in which there is a useful gain in power due to raising the Type I error rate. *Italicized *table cells indicate "high-ground" scenarios in which power is already high and raising the Type I error rate is unnecessary. Table cells that are neither bolded nor italicized indicate "low-ground" scenarios in which power is so low that raising the Type I error rate does not usefully boost power. A useful gain in power was defined as situations where raising the Type I error rate from 5% to 20% resulted in a 10% or greater gain in power, and power was above 70% when the Type I error rate was 20%.

### Effect of raising the Type 1 error rate

Table 1 also shows the effect of raising the Type I error rate on the asymptotic power of the Wald interaction test. Based on my criteria (10% gain in power and power above 70% when the Type I error rate is 20%), raising the Type I error rate from 5% to 20% resulted in a useful gain in power in only seven of the 27 interaction type/study size scenarios studied (26%). These "middle-ground" scenarios are the cells in Table 1 that are **bolded**.

The *italicized *scenarios in Table 1 are the eight situations (30%) where power was already adequate. In these "high-ground" situations, raising the Type I error rate to 20% will result in an appreciable increase in "false positives" with little or no real gain in power.

The unformatted (neither bolded nor italicized) scenarios in Table 1 represent the chronically underpowered ("low-ground") situations in which raising the Type I error rate achieves no useful gain in power. This occurred in twelve of the 27 interaction type/study size scenarios (44%).

Results of the simulations were almost identical to the asymptotic results for all study sizes. The simulation results confirmed that power for the Wald test for interaction, the likelihood ratio test for interaction and the Breslow-Day test of heterogeneity of the odds ratio, are essentially identical.

### Coverage of the confidence interval for β^3
 MathType@MTEF@5@5@+=feaafiart1ev1aaatCvAUfKttLearuWrP9MDH5MBPbIqV92AaeXatLxBI9gBaebbnrfifHhDYfgasaacH8akY=wiFfYdH8Gipec8Eeeu0xXdbba9frFj0=OqFfea0dXdd9vqai=hGuQ8kuc9pgc9s8qqaq=dirpe0xb9q8qiLsFr0=vr0=vr0dc8meaabaqaciaacaGaaeqabaqabeGadaaakeaaiiGacuWFYoGygaqcamaaBaaaleaacqaIZaWmaeqaaaaa@2F84@

Using the simulations, the coverage of the 95% confidence interval for the interaction term β^3
 MathType@MTEF@5@5@+=feaafiart1ev1aaatCvAUfKttLearuWrP9MDH5MBPbIqV92AaeXatLxBI9gBaebbnrfifHhDYfgasaacH8akY=wiFfYdH8Gipec8Eeeu0xXdbba9frFj0=OqFfea0dXdd9vqai=hGuQ8kuc9pgc9s8qqaq=dirpe0xb9q8qiLsFr0=vr0=vr0dc8meaabaqaciaacaGaaeqabaqabeGadaaakeaaiiGacuWFYoGygaqcamaaBaaaleaacqaIZaWmaeqaaaaa@2F84@ was computed. This is the proportion of 95% confidence intervals, from the 1000 simulations for each interaction type, that included the true (population) value for *β*_3 _(per Figure 1). Ideally, the coverage for a 95% confidence interval should be 95%. For all the interaction type/study size scenarios studied, the coverage was adequate, ranging from 93% to 96%. It was 93%–94% in 30% of scenarios, 95% in 44% of scenarios, and 96% in 26% of scenarios. This confirmed that the estimated standard error for β^3
 MathType@MTEF@5@5@+=feaafiart1ev1aaatCvAUfKttLearuWrP9MDH5MBPbIqV92AaeXatLxBI9gBaebbnrfifHhDYfgasaacH8akY=wiFfYdH8Gipec8Eeeu0xXdbba9frFj0=OqFfea0dXdd9vqai=hGuQ8kuc9pgc9s8qqaq=dirpe0xb9q8qiLsFr0=vr0=vr0dc8meaabaqaciaacaGaaeqabaqabeGadaaakeaaiiGacuWFYoGygaqcamaaBaaaleaacqaIZaWmaeqaaaaa@2F84@ tended to be consistently estimated by logistic regression across a range of interaction scenarios, even in the very small study size scenarios.

## Discussion

Two major points emerge from this analysis. First of all, it is striking that power for testing interaction (at the 5% level) is very low for several types of interaction, even in studies as large as 300 cases and 600 controls.

Second, these results call into question the wisdom of addressing the problem of low power for interaction tests by raising the Type I error rate. Power was so low in many of the scenarios studied (44%) that raising the Type I error rate failed to boost power to an acceptable level (defined as 70% in this study). In another 30% of scenarios, power was already above 90% at the 5% Type I error rate, so there was little benefit from raising the Type I error rate. In only about 1/4 of scenarios studied was there a useful gain in power due to raising the Type I error rate. Based on these data, I recommend investigators do not routinely raise the Type I error rate when assessing tests of interaction.

### Implications for epidemiologic practice

The implications of this study for epidemiologic practice depend in part on how individual investigators use tests of interaction. For investigators that see interaction tests as just one portion of an array of information to be utilized in the assessment of modification (along with using stratum-specific measures, confidence intervals, *apriori *hypotheses and biological knowledge), these results may be of limited importance, since these investigators do not use the interaction p-value as the sole basis for determining whether modification exists.

On the other hand, these results have considerable importance for investigators who rely on interaction tests as the sole basis for screening for potential interactions, and for investigators who rely almost exclusively on interaction tests to decide whether modification is present. Of particular concern are the "high ground" scenarios. If a study is already adequately powered for assessing interactions, due to a large study size, and the investigators are simultaneously screening many exposures and their interaction, then it will be very counterproductive to raise the Type I error rate, since this means that the number of spurious interactions detected by the test is increased without any tangible increase in the probability of detecting a true interaction.

### Limitations of multiplicative tests of interaction

I studied tests of interaction within a multiplicative model because they are the statistical procedure most commonly used to assess effect-measure modification. However, it is important to note that these interaction tests assess departure from perfect multiplicativity of effects, and therefore have profound limitations if the main interest lies in departure from additivity of effects [1,9,10].

### Study limitations

There are a number of limitations to this study. It assumed no confounding, no missing data, a fixed 1:2 case:control ratio, and studied a very specific situation – two binary exposures, each of 40% prevalence in the non-cases. Power will vary considerably with exposure prevalence, and would be higher if the variables were continuous, not binary. Further, only the logistic model was examined.

Perhaps the most profound limitation of this study is that it represents an application, to a data analysis situation, of the type of power criteria commonly used when designing a study. Thus, the choice of a 70% threshold for a useful gain in power could be criticized as unrealistic. However, even if the threshold was dropped from 70% to 50%, the strategy of raising the Type I error rate would result in a useful gain in power in only eleven of the 27 scenarios studied (41%).

## Conclusion

Investigators need to be aware that power for testing interactions is probably low in many epidemiologic studies. However, the results of this study suggest that routinely raising the Type I error rate for interaction tests is not an effective solution to the problem of the low power for tests of interaction. I recommend that investigators should not routinely raise the Type I error rate when assessing tests of interaction.

## Competing interests

The author(s) declare that they have no competing interests.

## Authors' contributions

The author conceived this study, conducted all analyses, and wrote the paper. He has read and approved the final manuscript.

## Appendix – Methodologic Details

### Type I and Type II Error Rate

The Type I error rate, or alpha, is the probability the study finds that an interaction between two exposures exists, when, in truth, there is no such interaction present in the population. This discrepancy arises because of sampling variability, i.e., by chance, the sample is a poor proxy for the population. The Type II error rate, or beta, is the probability that the study fails to detect an interaction between two exposures that, in truth, is present in the population. Power is 1 minus the Type II error rate. Raising the Type I error rate has the effect of decreasing the Type II error rate and thus increases the power of an interaction test.

### Wald Test for Interaction

The Wald test statistic for the test of interaction (see model 1) is:

β^32σ^β32
 MathType@MTEF@5@5@+=feaafiart1ev1aaatCvAUfKttLearuWrP9MDH5MBPbIqV92AaeXatLxBI9gBaebbnrfifHhDYfgasaacH8akY=wiFfYdH8Gipec8Eeeu0xXdbba9frFj0=OqFfea0dXdd9vqai=hGuQ8kuc9pgc9s8qqaq=dirpe0xb9q8qiLsFr0=vr0=vr0dc8meaabaqaciaacaGaaeqabaqabeGadaaakeaadaWcaaqaaGGaciqb=j7aIzaajaWaa0baaSqaaiabiodaZaqaaiabikdaYaaaaOqaaiqb=n8aZzaajaWaa0baaSqaaiab=j7aInaaBaaameaacqaIZaWmaeqaaaWcbaGaeGOmaidaaaaaaaa@3646@

The Wald test for interaction follows this power function [3]:

Power=F(−z1−α/2−|β3|σβ3)+1−F(z1−α/2−|β3|σβ3)
 MathType@MTEF@5@5@+=feaafiart1ev1aaatCvAUfKttLearuWrP9MDH5MBPbIqV92AaeXatLxBI9gBaebbnrfifHhDYfgasaacH8akY=wiFfYdH8Gipec8Eeeu0xXdbba9frFj0=OqFfea0dXdd9vqai=hGuQ8kuc9pgc9s8qqaq=dirpe0xb9q8qiLsFr0=vr0=vr0dc8meaabaqaciaacaGaaeqabaqabeGadaaakeaacqqGqbaucqqGVbWBcqqG3bWDcqqGLbqzcqqGYbGCcqGH9aqpcqWGgbGrdaqadaqaaiabgkHiTiabdQha6naaBaaaleaacqaIXaqmcqGHsisldaWcgaqaaGGaciab=f7aHbqaaiabikdaYaaaaeqaaOGaeyOeI0YaaSaaaeaadaabdaqaaiab=j7aInaaBaaaleaacqaIZaWmaeqaaaGccaGLhWUaayjcSdaabaGae83Wdm3aaSbaaSqaaiab=j7aInaaBaaameaacqaIZaWmaeqaaaWcbeaaaaaakiaawIcacaGLPaaacqGHRaWkcqaIXaqmcqGHsislcqWGgbGrdaqadaqaaiabdQha6naaBaaaleaacqaIXaqmcqGHsisldaWcgaqaaiab=f7aHbqaaiabikdaYaaaaeqaaOGaeyOeI0YaaSaaaeaadaabdaqaaiab=j7aInaaBaaaleaacqaIZaWmaeqaaaGccaGLhWUaayjcSdaabaGae83Wdm3aaSbaaSqaaiab=j7aInaaBaaameaacqaIZaWmaeqaaaWcbeaaaaaakiaawIcacaGLPaaaaaa@6109@

where *F *is the cumulative distribution function of the standard normal variate (*μ *= 0, *σ *= 1), *α *is the two-sided Type I error rate, and *β*_3 _is defined per model 1.

The Wald test statistic follows an approximate chi-square distribution under large sample conditions and the assumption of perfect multiplicativity of joint effects (i.e. assuming the null hypothesis). It has 1 degree of freedom in the situation of two binary exposures.

### Likelihood Ratio Test for Interaction

The likelihood ratio test can also be used to test *β*_3 _by comparing the log-likelihood for model (1) to the log-likelihood for the same model without the interaction term:

logit *p*_*x *_= *β*_0 _+ *β*_1_*A *+ *β*_2_*B *(model 2)

The likelihood ratio test for interaction is:

-2 [log-likelihood(model 2) - log-likelihood(model 1)]

Like the Wald test, this statistic is approximately chi-square distributed under large sample conditions and has 1 degree of freedom for two binary exposures.

### Breslow-Day Test

In contrast to these two model-based statistics (Wald and Likelihood Ratio), the Breslow-Day test for heterogeneity of the odds ratio is based on stratified analysis. The test statistic is:

∑h(nh11−E[nh11|ORMH])2var⁡[nh11|ORMH]
 MathType@MTEF@5@5@+=feaafiart1ev1aaatCvAUfKttLearuWrP9MDH5MBPbIqV92AaeXatLxBI9gBaebbnrfifHhDYfgasaacH8akY=wiFfYdH8Gipec8Eeeu0xXdbba9frFj0=OqFfea0dXdd9vqai=hGuQ8kuc9pgc9s8qqaq=dirpe0xb9q8qiLsFr0=vr0=vr0dc8meaabaqaciaacaGaaeqabaqabeGadaaakeaadaaeqbqaamaalaaabaWaaeWaaeaacqWGUbGBdaWgaaWcbaGaemiAaGMaeGymaeJaeGymaedabeaakiabgkHiTiabdweafnaadmaabaGaemOBa42aaSbaaSqaaiabdIgaOjabigdaXiabigdaXaqabaGccqGG8baFcqWGpbWtcqWGsbGudaWgaaWcbaGaemyta0KaemisaGeabeaaaOGaay5waiaaw2faaaGaayjkaiaawMcaamaaCaaaleqabaGaeGOmaidaaaGcbaGagiODayNaeiyyaeMaeiOCai3aamWaaeaacqWGUbGBdaWgaaWcbaGaemiAaGMaeGymaeJaeGymaedabeaakiabcYha8jabd+eapjabdkfasnaaBaaaleaacqWGnbqtcqWGibasaeqaaaGccaGLBbGaayzxaaaaaaWcbaGaemiAaGgabeqdcqGHris5aaaa@5802@

where *n*_*h*11 _is the count for the doubly unexposed cell of the *h*^th ^level of the stratification variable, *OR*_*MH *_is the Mantel-Haenszel odds ratio estimate, and *h *= 2 if both exposures are binary. Like the other two tests, it has an approximate chi-square distribution under large sample conditions.
